# Genetic compensation triggered by actin mutation prevents the muscle damage caused by loss of actin protein

**DOI:** 10.1371/journal.pgen.1007212

**Published:** 2018-02-08

**Authors:** Tamar E. Sztal, Emily A. McKaige, Caitlin Williams, Avnika A. Ruparelia, Robert J. Bryson-Richardson

**Affiliations:** School of Biological Sciences, Monash University, Melbourne, Victoria Australia; Max-Planck-Institut fur Herz- und Lungenforschung W G Kerckhoff-Institute, GERMANY

## Abstract

The lack of a mutant phenotype in homozygous mutant individuals’ due to compensatory gene expression triggered upstream of protein function has been identified as genetic compensation. Whilst this intriguing process has been recognized in zebrafish, the presence of homozygous loss of function mutations in healthy human individuals suggests that compensation may not be restricted to this model. Loss of skeletal α-actin results in nemaline myopathy and we have previously shown that the pathological symptoms of the disease and reduction in muscle performance are recapitulated in a zebrafish antisense morpholino knockdown model. Here we reveal that a genetic *actc1b* mutant exhibits mild muscle defects and is unaffected by injection of the *actc1b* targeting morpholino. We further show that the milder phenotype results from a compensatory transcriptional upregulation of an actin paralogue providing a novel approach to be explored for the treatment of actin myopathy. Our findings provide further evidence that genetic compensation may influence the penetrance of disease-causing mutations.

## Author summary

Many healthy individuals carry loss of function mutations in essential genes that would normally be deleterious for survival. Intriguingly, it may be the presence of the genomic lesion itself in these individuals that triggers the compensatory pathways. It is not known how widespread this phenomenon is in vertebrate populations and how genetic compensation is activated. We have shown that knockdown of actin causes nemaline myopathy as indicated by the formation of nemaline bodies within the skeletal muscle and reduced muscle function which, remarkably, we did not observe in an actin genetic mutant. We have identified that protection from the disease phenotype results from transcriptional upregulation of an actin paralogue restoring actin protein in the skeletal muscle. This study demonstrates that genetic compensation may be more prevalent than previously anticipated and highlights phenotypic differences resulting from genetic mutations versus antisense knockdown approaches. Furthermore, we suggest that activating compensatory pathways may be exploited as a potential novel therapeutic approach for human disorders caused by loss of function mutations.

## Introduction

Genetic compensation exists as a mechanism to buffer the organism against gene loss that would otherwise be deleterious to survival. Whilst this term has been used to describe dosage compensation, evolution resulting in reversion to ancestral phenotypes, and gene duplication compensating for mutation, in the present study we refer to genetic compensation as altered gene expression resulting in a normal phenotype in a homozygous mutant individual. Studies in plants [1], worms [2], and yeast [3,4] have demonstrated that genetic robustness can be achieved by the presence of duplicate gene copies, which have retained a similar biological function, or through the presence of redundant pathways. A single case of genetic compensation in zebrafish has been shown to result in the activation of a network of gene expression resulting from the presence of a mutation in *egf17*, but not from gene knockdown [5]. Intriguingly, data from widespread application of whole genome sequencing demonstrates that homozygous mutations predicted to cause a loss of function, that would normally cause disease, may be present in healthy individuals in the human population [6], suggesting that compensation may be more widespread than previously thought. However, the lack of functionally characterized examples raise the question of whether this is an isolated case, or if genetic compensation may be a common mechanism contributing to genetic robustness in vertebrates.

In the skeletal muscle, expression of cardiac muscle α-actin (ACTC1) can partially compensate for loss of ACTA1 to ameliorate the loss of function phenotype [7]. α-actin is an essential component of the thin filament, with its mutation resulting in a range of skeletal muscle disorders, including nemaline myopathy [8]. ACTA1 differs from ACTC1 by only four amino acids and both are co-expressed in the skeletal muscle and heart during development [9–11]. In vertebrates, ACTC1 is the predominant actin isoform in fetal skeletal muscle, however, after birth *ACTC1* is down regulated [11] and by adulthood it comprises <5% of adult skeletal muscle [12], with ACTA1 becoming the predominant form.

Most ACTA1 mutations are de novo dominant mutations with only 10% of patients’ carrying recessively inherited loss of function mutations [8]. Patients displaying a complete absence of ACTA1 typically show retention of ACTC1 in their skeletal muscle, with the level of retention determining the level of clinical severity [7]. Transgenic overexpression of ACTC1 was also shown to rescue the early lethality observed in recessive *ACTA1*^*-/-*^ knockout mice strains [13], further demonstrating the potential for the levels of ACTA1 paralogues to influence disease severity.

Here we uncover a novel case of genetic compensation in zebrafish within the highly conserved actin gene family. We [14] and others [15,16] have shown that loss or knockdown of skeletal α-actin is catastrophic for muscle structure and function. Antisense Morpholino (MO) knockdown of Actc1b results in the formation of nemaline bodies and reduced skeletal muscle performance [14]. In contrast, we observe very mild defects in a genetic *actc1b* mutant. We determine that this is due to a compensatory transcriptional upregulation of an α-actin paralogue in the skeletal muscle buffering the loss of Actc1b function. Our study not only supports the existence of genetic compensation as a phenomenon affecting phenotypic diversity in vertebrates, but also identifies a genetic process that may have therapeutic implications for nemaline myopathy and other actin-related diseases.

## Results

### *actc1b* is the predominantly expressed α-actin in zebrafish skeletal muscle

**Fig 1:**
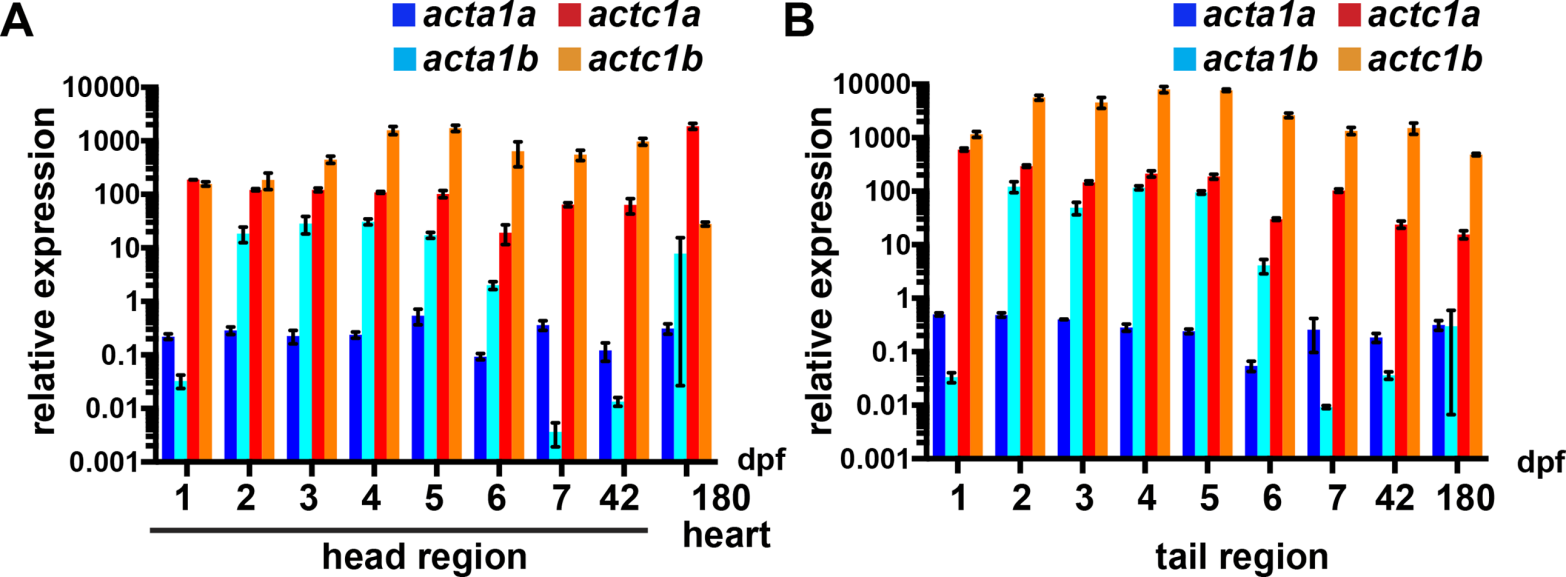
Quantitative RT-PCR analysis for zebrafish α-actin genes. mRNA expression of *acta1a*, *acta1b*, *actc1a*, and *actc1b* genes in the A) head (comprising the heart tissue) and B) tail (comprising predominantly skeletal muscle tissue) during zebrafish development. Error bars for A) and B) represent SEM for three independent biological replicates each consisting of a pooled sample of 20–30 embryos.

To investigate α-actin function in zebrafish we initially examined the expression of the α-actin paralogues. Four α-actin genes (*acta1a*, *acta1b*, *actc1a*, and *actc1b*) have been identified in the zebrafish [14], and all are expressed in both skeletal and cardiac muscle during early zebrafish development (S1 Fig). qRT-PCR analyses showed that, whilst all genes are expressed during early embryogenesis, *actc1b* is expressed at much higher levels than the other paralogues and that by 180 dpf (days post-fertilization) *actc1a* and *actc1b* are the predominant α-actin isoforms in the heart and skeletal muscle respectively (Fig 1, S1 Table, and S2 Table).

We were surprised to find that the two cardiac α-actin genes were the most highly expressed in the muscle and therefore analyzed the sequence similarity between the α-actin isoforms. An analysis of the surrounding genomic regions shows conserved synteny between human *ACTA1* and zebrafish *acta1a* and *acta1b* genes, and similarly between human *ACTC1* and zebrafish *actc1a* and *actc1b* genes (S2 Fig) suggesting that indeed the zebrafish genes are orthologous to their respective human genes. Interestingly, a maximum likelihood phylogenetic analysis predicts that, following an initial duplication, the *ACTA1* and *ACTC1* copies separate into distinct clades except in zebrafish where *actc1a*, *acta1b*, *actc1a*, and *actc1b* all form a clade together with *ACTA1* (S3 Fig). Although this suggests that the zebrafish *actc1* genes may have evolved to become more similar to *ACTA1* than to their respective orthologue, the low bootstrapping values reflect a very high level of nucleotide sequence conservation between all genes ranging from 85–90% identity.

### Genetic mutation in *actc1b* causes a mild skeletal muscle phenotype

**Fig 2:**
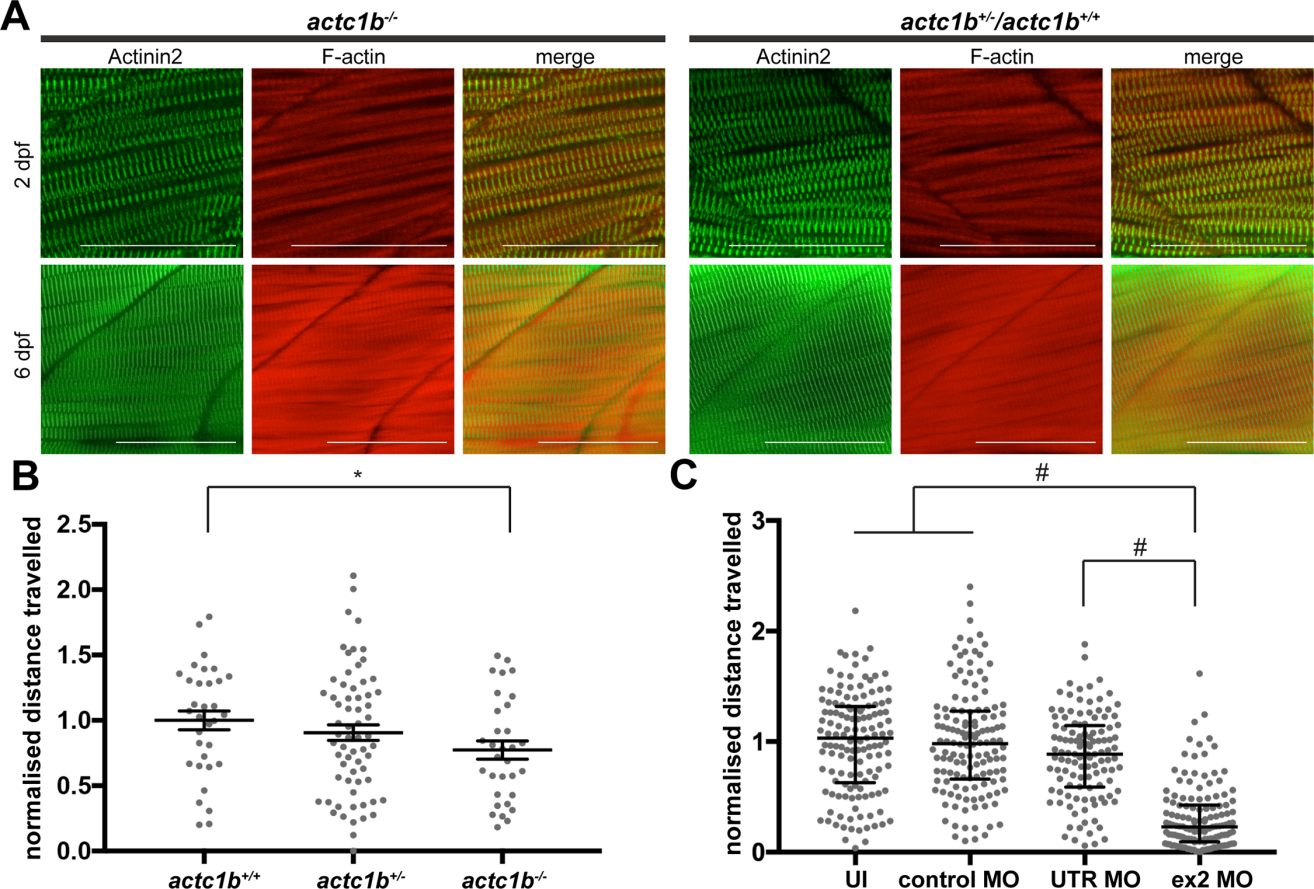
Characterization of muscle phenotypes in *actc1b*^*-/-*^ mutants and Actc1b morphants. A) Antibody labelling against Actinin2 and Phalloidin of *actc1b*^*-/-*^ mutants and wildtype siblings with Actinin2 (green) and F-actin (red) at 2 dpf and 6 dpf showing normal muscle morphology. Scale bar represents 50μm. B) Locomotion assays show a significant reduction in distance travelled by *actc1b*^*-/-*^ mutants compared to siblings (*actc1b*^*+/-*^ and *actc1b*^*+/+*^) zebrafish. Error bars represent SEM for three independent experiment (n = 6,11,16 for *actc1b*^*+/+*^; n = 24,23,18 for *actc1b*^*+/-*^; and n = 13,9,10 for *actc1b*^*-/-*^ per experiment), *p<0.05 using a one-way ANOVA. C) Locomotion assays showing a significant reduction in distance travelled by Actc1b ex2 and UTR morphants compared to both Standard Control MO injected and uninjected zebrafish. No significant difference in locomotion is observed for Standard Control MO injected and uninjected zebrafish. Error bars represent median values and interquartile range (pooled samples from 3 independent experiments n = 45,48,46 for Actc1b ex2 MO; n = 45,48,33 for Actc1b UTR MO; n = 45,48,47 for Standard Control MO; and n = 48,47,45 for uninjected zebrafish), #p<0.0001 using a Kruskal-Wallis Test.

To analyze Actc1b function in zebrafish we examined the phenotype of *actc1b* mutants (*actc1b*^*sa12367*^, referred to hereafter as *actc1b*^*-/-*^). This mutant was generated using ENU mutagenesis and the presence of a nonsense mutation at amino acid 5 was verified by competitive allele specific PCR (KASP) genotyping [17]. We verified the mutation in the *actc1b*^*-/-*^ mutant strain and confirmed that the MO binding sites were intact (S4 Fig). Surprisingly, we found no difference in the appearance of the muscle fibers between *actc1b*^*-/-*^ embryos and their wildtype siblings (Fig 2A) and only a small, but significant, reduction in swimming capabilities of *actc1b*^*-/-*^ embryos compared to wildtype siblings (*actc1b*^*+/+*^ mean 1.0±0.1SEM and *actc1b*^*-/-*^ mean 0.77±0.12SEM; p 0.0128; Fig 2B). Whilst *actc1b*^*-/-*^ fish show only a 23% reduction in distance swum, in line with our previous work, Actc1b ex2 morphants show a dramatic reduction in swimming performance compared to Standard Control morphants, UTR MO morphants, and uninjected controls (median values Actc1b ex2 MO 0.23, UTR MO 0.89, Standard Control MO 0.98, and uninjected 1.03; p<0.0001 for ex2 MO against all other conditions; Fig 2C).

**Fig 3:**
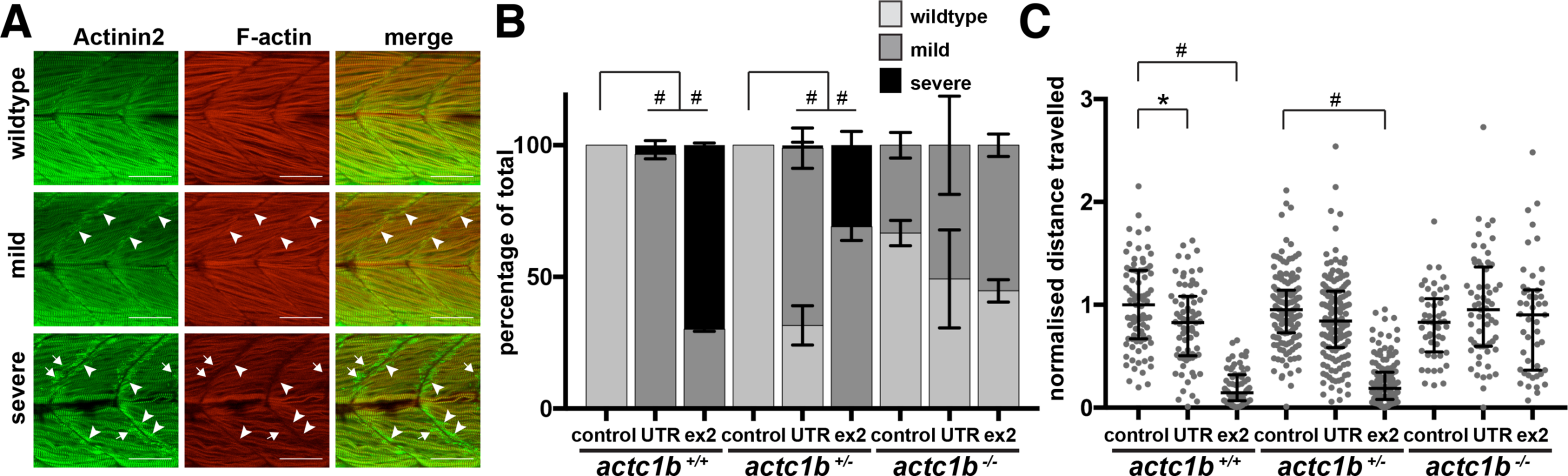
Characterization of phenotypic severity following Actc1b morpholino knockdown. A) *actc1b*^*-/-*^ and wildtype siblings (*actc1b*^*+/-*^ and *actc1b*^*+/+*^) injected with either an Actc1b ex2, Actc1b UTR or Standard Control morpholino were stained with Actinin2 (green) and F-actin (red) and phenotypes were scored as either wildtype, mild (small outgrowth of aggregates at the myosepta (arrowheads)) or severe (large outgrowth of aggregates at the myosepta (arrowheads) and Actinin2 positive aggregates throughout the muscle fibers (arrows)). Scale bar represents 50μm. B) Quantification of phenotypic severity for *actc1b*^*-/-*^ and wildtype siblings injected with Actc1b ex2 and Actc1b UTR MOs compared to Standard Control MO injected zebrafish. Error bars represent SEM for three independent experiments (for Actc1b ex2 MO: n = 26,14,16 *actc1b*^*+/+*^, n = 31,35,29 *actc1b*^*+/-*^ and n = 12,11,23 *actc1b*^*-/-*^, for Actc1b UTR MO: n = 23,21,17 *actc1b*^*+/+*^, n = 28,30,23 *actc1b*^*+/-*^ and n = 8,11,8 *actc1b*^*-/-*^ and for Standard Control MO: n = 11,8,10 *actc1b*^*+/+*^, n = 11,13,11 *actc1b*^*+/-*^ and n = 9,12,4 *actc1b*^*-/-*^), *#*p<0.0001 using a Chi-square test. C) Locomotion assays show a significant reduction in distance travelled by *actc1b*^*+/+*^ injected with an Actc1b UTR MO and Actc1b ex2 MO compared to Standard Control MO, using a Kruskal-Wallis Test. Locomotion assays show a significant reduction in distance travelled by *actc1b*^*+/-*^ injected with an Actc1b ex2 MO compared to Control MO, using a Kruskal-Wallis Test. No difference in distance travelled is observed between *actc1b*^*-/-*^ mutants injected with either an Actc1b UTR MO, Actc1b ex2 MO or Standard Control MO. Error bars represent median values with interquartile range (pooled samples from 3 independent experiments for Actc1b ex2 MO: n = 25,21,20 *actc1b*^*+/+*^, n = 41,53,49 *actc1b*^*+/-*^ and n = 20,14,15 *actc1b*^*-/-*^, for Actc1b UTR MO: n = 30,24,19 *actc1b*^*+/+*^, n = 42,47,57 *actc1b*^*+/-*^ and n = 21,23,17 *actc1b*^*-/-*^ and for Standard Control MO: n = 31,28,26 *actc1b*^*+/+*^, n = 41,50,52 *actc1b*^*+/-*^ and n = 21,14,17 *actc1b*^*-/-*^). *p<0.05 and #p<0.0001.

To determine whether the phenotypic differences observed were due to MO off-target effects we injected the Actc1b ex2, UTR MO, or Standard Control MO into an incross of *actc1b*^*+/-*^ zebrafish and assessed phenotypic severity in their offspring in three independent experiments. The phenotypes were classed as either wildtype, mild (slight disruption to the muscle fibers), or severe (large disruption to the muscle fibers and Actinin2 aggregates at the myosepta) (Fig 3A). Remarkably, we found that *actc1b*^*-/-*^ mutants never displayed a severe phenotype when injected with either the Actc1b ex2 MO (21 wildtype, 25 mild, 0 severe) or Actc1b UTR MO (14 wildtype, 13 mild, 0 severe) similar to the Standard Control MO (16 wildtype, 9 mild, 0 severe). However, severe phenotypes were observed in *actc1b*^*+/+*^ and *actc1b*^*+/-*^ siblings injected with either Actc1b MO (*actc1b*^*+/+*^ injected with the Actc1b ex2 MO: 0 wildtype, 2 mild, 39 severe or Actc1b UTR MO: 0 wildtype, 59 mild, 17 severe and for *actc1b*^*+/-*^ injected with the Actc1b ex2 MO: 0 wildtype, 66 mild, 29 severe or Actc1b UTR MO: 25 wildtype, 55 mild, 1 severe) which were not observed following injection with the Standard Control MO (for *actc1b*^*+/+*^: 29 wildtype, 0 mild, 0 severe and for *actc1b*^*+/-*^: 35 wildtype, 0 mild, 0 severe) (Fig 3B). The change in proportions of phenotype classes was significant for both morpholinos into *actc1b*^*+/+*^ and *actc1b*^*+/-*^ compared to control MO (p<0.0001, Chi-square test). The insensitivity of the *actc1b*^*-/-*^ mutants to Actc1b MO knockdown demonstrates that the severe phenotypes are not due to off-target effects.

In addition, we measured the locomotion of *actc1b*^*-/-*^ mutants and their wildtype siblings injected with either the Actc1b ex2, Actc1b UTR, or Standard Control MO at 6 dpf. We observed a significant reduction in distance travelled by *actc1b*^*+/+*^ and *actc1b*^*+/-*^ siblings injected with an Actc1b ex2 MO (median 0.14 for *actc1b*^*+/+*^, p<0.0001; and 0.19 for *actc1b*^*+/-*^, p<0.0001) and for *actc1b*^*+/+*^ siblings injected with an Actc1b UTR MO (median 0.83, p0.046) compared to the Standard Control MO (1.0 for *actc1b*^*+/+*^ and 0.95 for *actc1b*^*+/-*^) (Fig 3C). In contrast, *actc1b*^*-/-*^ mutants injected with either an Actc1b ex2 (median 0.90) or an Actc1b UTR MO (median 0.95) show comparable locomotion to those injected with a Standard Control MO (median 0.83) confirming that *actc1b*^*-/-*^ mutants are indeed unaffected by Actc1b knockdown (Fig 3C).

### *actc1b* genetic mutants display normal actin levels

**Fig 4:**
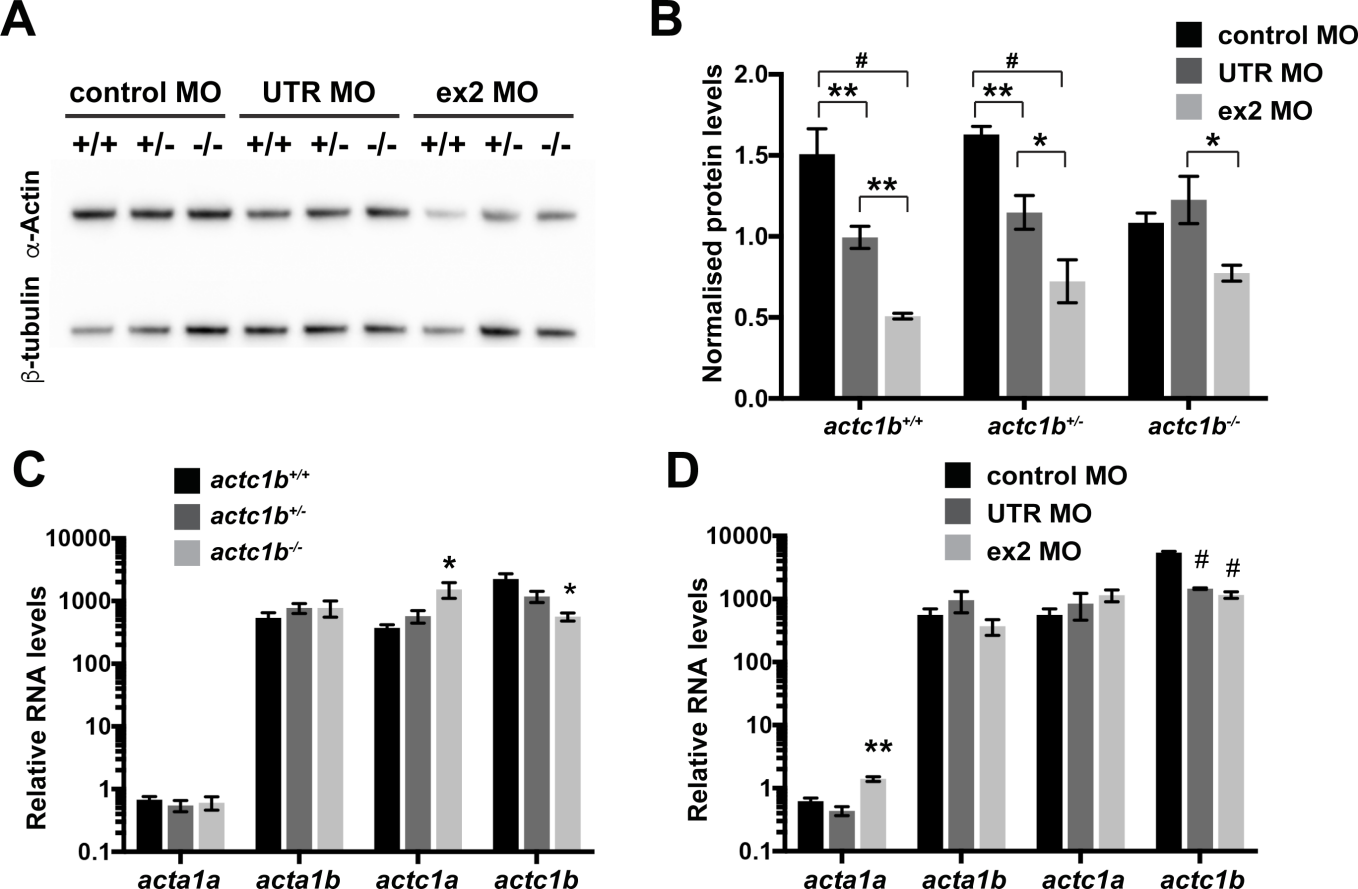
Quantification of RNA and protein levels following Actc1b morpholino knockdown. A) Representative data from western blot analysis for α-actin protein expression in *actc1b*^*-/-*^ and their wildtype siblings (*actc1b*^*+/-*^ and *actc1b*^*+/+*^) at 2 dpf injected with either an Actc1b UTR, Actc1b ex2, or Standard Control MO. β-tubulin was used as a loading control. B) Quantification of western blot analysis from three independent replicate experiments of A) (S5 Fig) consisting of a pooled sample of 20 tails whereby α-actin protein levels were normalized against the -tubulin loading control. Error bars represent SEM for three independent experiments, *p<0.05, **p<0.01, and #p<0.0001 using a two-way ANOVA. C) Quantitative RT-PCR analysis for zebrafish *acta1a*, *acta1b*, *actc1a*, and *actc1b* genes in tail samples from *actc1b*^*+/+*^, *actc1b*^*+/-*^, and *actc1b*^*-/-*^ at 2 dpf. Error bars represent SEM for three independent experiments each consisting of a pooled sample of 30 tail samples, *p<0.05 indicates difference from *actc1b*^*+/+*^ using a one-way ANOVA. D) Quantitative RT-PCR analysis for zebrafish *acta1a*, *acta1b*, *actc1a*, and *actc1b* genes in whole embryos from Actc1b ex2 and Actc1b UTR morphants compared to Standard control MO injected zebrafish at 2 dpf. Error bars represent SEM for three independent experiments each consisting of a pooled sample of 20–30 embryos, **p<0.01, and #p<0.001 indicate difference from control MO using a one-way ANOVA.

To determine why there was not a loss of function phenotype in *actc1b*^*-/-*^ mutants we first measured α-actin levels in the skeletal muscle of *actc1b*^*-/-*^ mutants, wildtype siblings, and Actc1b morphants by western blot analysis. As previously observed (Sztal et al, 2015), Actc1b morphants display decreased total actin levels compared to siblings injected with a control morpholino (Fig 4A and S5 Fig). Quantification and analysis of western blots by two-way ANOVA shows that *actc1b*^*+/+*^ and *actc1b*^*+/-*^ siblings injected with either an Actc1b UTR MO (mean 0.94 SD 0.19 for *actc1b*^*+/+*^ (p0.0048) and mean 1.15 ±0.18SD for *actc1b*^*+/-*^ (p0.0077)) or Actc1b ex2 MO (mean 0.51 ±0.03SD for *actc1b*^*+/+*^ (p<0.0001) and mean 0.72 ±0.23SD for *actc1b*^*+/-*^ (p<0.0001)) show significantly reduced α-actin compared to Control MO injected *actc1b*^*+/+*^ and *actc1b*^*+/-*^ siblings (mean 1.51 ±0.27SD for *actc1b*^*+/+*^and mean 1.63 ±0.09SD for *actc1b*^*+/-*^) (Fig 4B and S5 Fig). However, *actc1b*^*-/-*^ mutants injected with either an Actc1b UTR MO (mean 1.23 ±0.25SD) or Actc1b ex2 MO (mean 0.77 ±0.09SD), show no decrease in α-actin levels compared to or Standard Control MO (mean 1.08 ±0.11SD; p0.5779 and p0.0955 respectively) explaining the lack of phenotype compared to *actc1b*^+/+^ and *actc1b*^*+/-*^ morphants. The analysis also identified a significant interaction between genotype and MO treatment (p0.0088) confirming a genotype specific effect of the morpholinos.

### Transcriptional upregulation of *actc1a* compensates for the loss of actc1b

Given the normal levels of actin in *actc1b*^-/-^ fish we hypothesized that other α-actin paralogues were compensating for the loss of Actc1b. To determine the expression of the four α-actin paralogues we assayed the skeletal muscle of *actc1b*^*+/+*^, *actc1b*^*+/-*^, and *actc1b*^*-/-*^ embryos using qRT-PCR. We found a significant decrease in RNA levels of *actc1b* in Actc1b morphants (UTR MO mean 1457 ±70SD, ex2 MO mean 1160 ±237SD, compared to Standard Control MO mean 5418 ±505SD, p<0.0001 for both) as previously shown in Sztal et al (2015) as well as in *actc1b*^*-/-*^ mutants (mean 563 ±142SD compared to *actc1b*^*+/+*^ mean 2243 ±848SD, p0.0172), suggesting the *actc1b* gene product is degraded by nonsense mediated decay (Fig 4C & 4D). Interestingly, *actc1a* expression was significantly increased in *actc1b*^*-/-*^ mutants compared to *actc1b*^*+/+*^ siblings (mean 15334 ±742SD and mean 374 ±80SD respectively, p0.0346, Fig 4C). Conversely, Actc1b morphants did not display significant changes in the expression of any of the other α-actin isoforms compared to siblings injected with a Standard Control MO (Fig 4D).

To confirm that the compensatory upregulation of *actc1a* was responsible for the milder phenotype in *actc1b*^*-/-*^ mutants, we reasoned that if Actc1a was reduced in *actc1b*^*-/-*^ mutants, they would display a more severe phenotype, comparable to Actc1b morphants. We used a MO targeting the splice donor site of exon 2 (Actc1a ex2 MO) to knockdown Actc1a. To determine a subphenotypic dose for the Actc1a ex2 MO we injected 0.5, 1.0, or 2.0ng of MO into wildtype embryos and performed both RT-PCR and western analyses to determine MO efficiency. We were able to detect a small decrease in α-actin by western blot and RT-PCR analyses revealed two amplicons, a smaller product observed in all of the samples including controls which was reduced in the MO injected embryos, and a larger band, only observed in 1.0 and 2.0ng injected samples (S6A Fig). We sequenced the larger band and confirmed that it corresponds to the inclusion of intron 2, resulting from mis-splicing of exon 2 and 3, causing the addition of three amino acids and a stop codon which would undoubtedly disrupt Actc1a function. Mutations in *actc1a* have been shown to cause heart defects resulting in decreased cardiac contractility and altered blood flow [18]. Although the skeletal muscle appeared unaffected by MO injections (S6E Fig), we observed a slightly dilated heart in embryos injected with a 1ng MO concentration which became more severe as the MO dose increased (S6D Fig). Based on these observations, we selected a 1ng MO dose to use in further experiments.

**Fig 5:**
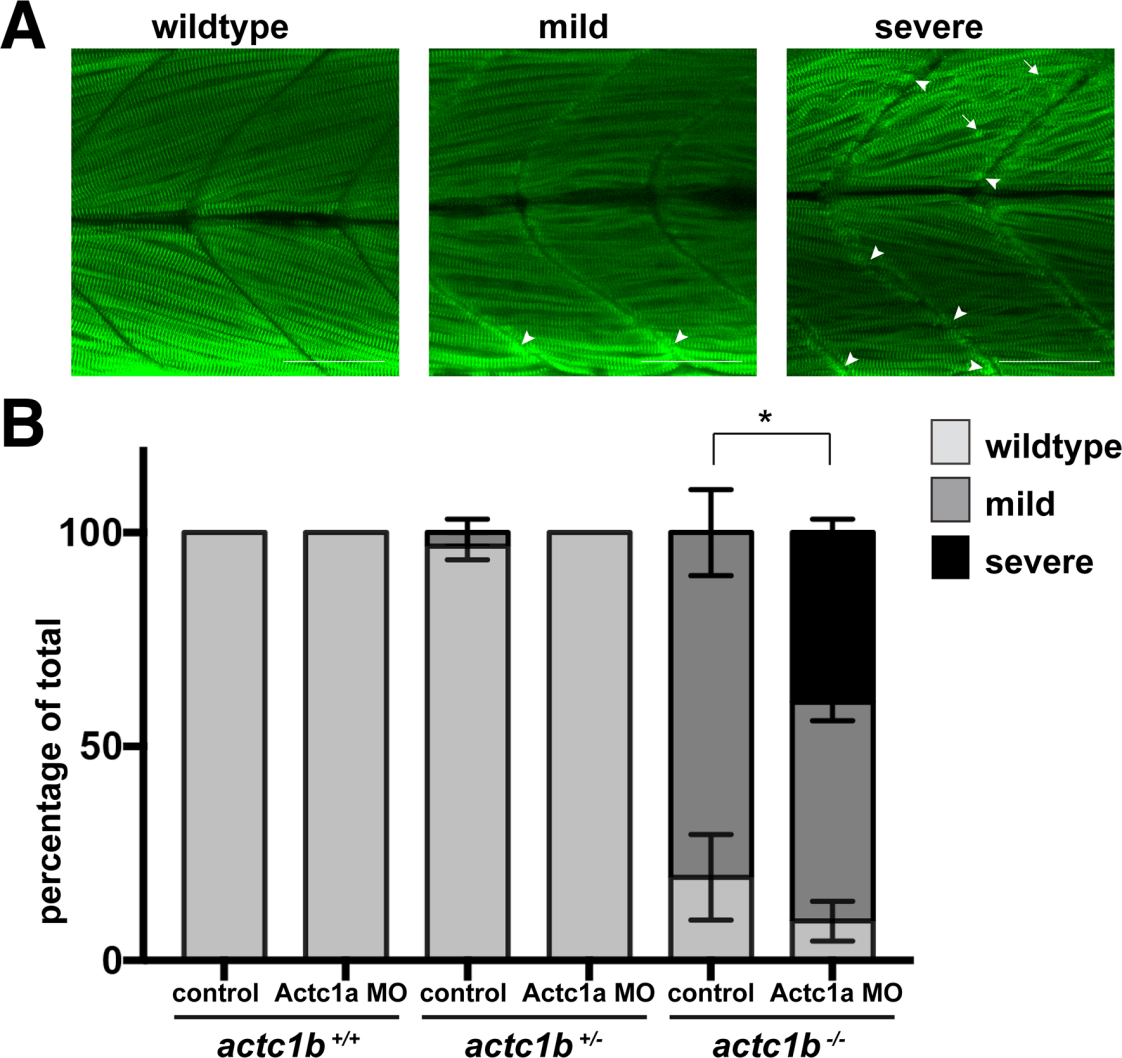
Characterization of phenotypic severity following Actc1a morpholino knockdown. A) *actc1b*^*-/-*^ and wildtype siblings (*actc1b*^*+/-*^ and *actc1b*^*+/+*^) injected with either an Actc1a splice or Standard Control MO were stained with Actinin2 and phenotypes were scored as either wildtype, mild (small outgrowth of aggregates at the myosepta (arrowheads)) or severe (large outgrowth of aggregates at the myosepta (arrowheads) and Actinin2 positive aggregates throughout the muscle fibers (arrows)). Scale bar represents 50μm. B) Quantification of the phenotypic severity for *actc1b*^*-/-*^ and wildtype siblings (*actc1b*^*+/+*^ and *actc1b*^*+/-*^) injected with Actc1a splice compared to Standard Control MO injected zebrafish. Error bars represent SEM for three independent experiments (for Actc1a MO n = 8,13,7 *actc1b*^*+/+*^, n = 27,16,16 *actc1b*^*+/-*^ and n = 7,15,7 *actc1b*^*+/+*^ and for Standard Control MO n = 7,3,7 *actc1b*^*+/+*^, n = 16,21,20 *actc1b*^*+/-*^ and n = 4,8,6 *actc1b*^*-/-*^), *p<0.05 indicates a significant difference in phenotype proportions using a Chi-square test.

We then injected the Actc1a ex2 MO (or corresponding dose of a Standard Control MO) into an incross of *actc1b*^*+/-*^ zebrafish and assessed phenotypic severity in their offspring in three independent experiments. The phenotypes were classed as either wildtype, mild (slight disruption to the muscle fibers), or severe (large disruption to the muscle fibers and Actinin2 aggregates at the myosepta) (Fig 5A). In *actc1b*^*+/+*^ and *actc1b*^*+/-*^ siblings injected with either the Standard Control (*actc1b*^*+/+*^: 17 wildtype, 0 mild, 0 severe and *actc1b*^*+/-*^: 55 wildtype, 2 mild, 0 severe) or Actc1a MO (*actc1b*^*+/+*^: 28 wildtype, 0 mild, 0 severe and *actc1b*^*+/-*^: 59 wildtype, 0 mild, 0 severe) we only observed a wildtype or mild phenotype. However, when we injected the Actc1a MO into *actc1b*^*-/-*^ mutants we observed a severe phenotype in approximately 40% of fish (3 wildtype, 15 mild, 11 severe) which was not observed in Standard Control MO injected *actc1b*^*-/-*^ mutants (4 wildtype, 14 mild, 0 severe, change in phenotype proportions p0.0106 Chi-square test). Taken together these results demonstrate that upregulation of the *actc1a* paralogue is protective in *actc1b*^*-/-*^ mutants.

## Discussion

We have identified compensation triggered by a mutation in *actc1b* but not following morpholino mediated knockdown of Actc1b. There has been considerable debate recently as a result of phenotypic differences between mutant lines and morpholino-mediated knockdown [19–22]. The study by Rossi et al (2015) provided an example where this difference in phenotype was due to compensation, rather than morpholino off target effects as previously suggested. The data presented in the current study identifies another example of the phenomenon of genetic compensation, and, rather than compensation in *egf17* mutants being an isolated case, suggests that this process may be more widespread. It also suggests that in some cases, rather than the phenotypic differences between mutant and knockdown animals being due to off-target or non-specific effects, genetic compensation may influence the phenotypic penetrance of deleterious mutations. Morpholino knockdown may therefore potentially reveal the phenotype resulting from reduction of the protein, without any compensatory transcription upregulation of paralogues or alternative pathways [23].

In zebrafish *actc1a* is expressed in the skeletal muscle during early embryogenesis, but is downregulated in the skeletal muscle as development proceeds. However, upregulation of *actc1a* is sufficient to compensate for the loss of Actc1b mimicking the upregulation of ACTC1 in patients suffering from recessive nemaline myopathy caused by mutations in *ACTA1* [7]. In this situation, patients have a complete absence of skeletal α-actin but instead cardiac α-actin is upregulated leading to a milder disease phenotype than patients with dominant mutations in *ACTA1* [7,15]. The levels of cardiac α-actin in these patients determines the clinical severity of the disease [7].

In contrast to the compensation we have identified, the specific response in the skeletal muscle in individuals with recessive ACTA1 mutations is not sufficient to prevent disease and the majority of individuals die within 6 months of birth [7]. Transgenic expression of ACTC1 in the skeletal muscle is, however, able to rescue both recessive *ACTA1*^*-/-*^ and dominant ACTA1^D286G^ mutations in mice [13,16], consistent with our findings that upregulation of actin paralogues can prevent a disease phenotype. The absence of a similar compensatory mechanism resulting from reduction of Actc1b following morpholino antisense mediated knockdown suggests that compensation is not induced by the reduction in Actc1b, but at a step prior to protein formation.

While the mechanisms of genetic compensation remain unclear, two different models have been recently proposed suggesting that compensation may be activated through either a DNA damage response or by the degradation of mutant RNA and subsequent activation of common microRNAs or ribosomal binding proteins to stabilize compensatory interactions [24]. In the case of *actc1b*, we have shown that there are no alternative transcripts produced in *actc1b*^*-/-*^ mutants and that the resulting mRNA is likely to be non-functional and degraded by nonsense mediated decay, which may activate compensation. However, we cannot rule out the possibility that it is either the DNA lesion itself, or the presence of the mutant mRNA that may trigger compensation. Recent studies have shown that a missense mutation in *actc1a* (*actc1a*^*s434*^) causes severe defects in cardiac contractility and altered blood flow resulting from the loss of polymerized cardiac actin [18]. Injection of an Actc1a MO mimicked the heart edema and lack of endocardial cushion formation observed in *actc1a* mutants [18] suggesting that compensation does not play a role in *actc1a*^*s434*^ mutants. However, it may be that if an alternative mutation, such as a nonsense mutation, is introduced or nonsense mediated pathways are activated that compensatory α-actin paralogues responses would be induced. Nevertheless, identifying the specific factors that trigger the compensatory upregulation of cardiac α-actin in the skeletal muscle tissues is the next challenge and could have therapeutic applications to ameliorate ACTA1 skeletal muscle diseases.

Genetic robustness against null mutations appears to be a universal phenomenon in all organisms, however, the mechanisms determining compensation may differ. The existence of duplicate gene copies to compensate for the loss of an essential gene has been previously observed in yeast [4] and worms [2] with genome-wide deletion experiments revealing a significantly lower percentage of duplicates compared to singletons are essential for viability and fertility. Inherited mutations have also been shown to have variable consequences in different individuals [25], which may be due to a plasticity of genetic compensatory responses masking the phenotypic effect of deleterious alleles. Our study verifies the existence of compensatory mechanisms, leading to a milder phenotype in ACTA1 recessive myopathy. More importantly, we suggest that similar compensatory responses may underline phenotypic differences in disease penetrance in the human condition.

## Materials and methods

### Ethics statement

Fish maintenance and handling was carried out as per the standard operating procedures approved by the Monash Animal Services Animal Ethics Committee under breeding colony license MARP/2015/004/BC. Fish were anaesthetized using Tricaine methanesulfonate.

### Zebrafish maintenance, morpholino injections and genotyping

Zebrafish were maintained according to standard protocols [26]. The Actc1b ex2 (5’ TGCAGTGTTTTTTTCACCTGGTGAC 3’) Actc1b UTR (5’ GGTCAAGTTGTTATCACAAGACTGA 3’), Actc1a ex2 (5’ TACATGCTTTAGAAGCCCACCTGGT 3’) and Standard Control (5’ CCTCTTACCTCAGTTACAATTTATA 3’) MOs (GeneTools) were diluted in distilled water and co-injected with Cascade Blue labeled dextran (Molecular Probes) into one- to two-cell embryos MO concentrations were calibrated according to [27] at the indicated amounts (2.0 ng for the Actc1b ex2 and UTR MOs corresponding to a concentration of 0.5mM; 0.5, 1.0, or 2.0ng for the Actc1a ex2 MO corresponding to concentrations of 0.125mM, 0.25mM, and 0.5mM; and 1.0 or 2.0ng for the Standard Control MO corresponding to concentrations of 0.25mM and 0.5mM). At 1 dpf, the embryos were sorted for Cascade Blue labeling. The *actc1b* mutant line (sa12367) was obtained from the Zebrafish International Resource Centre [17]. Allele specific PCR KASP technology (Geneworks) was used for genotyping.

### *In situ* hybridization

Whole-mount *in situ* hybridization was carried out as described previously [28]. Probes were constructed using specific gene primers (*acta1a* F: CAACATCCTATCATTGCCTCCT and R: CATGTTCAGTTTTATTTGTCTGTTGA; *acta1b* F: ATTCATCGGCTGCATCTGTC and R: TTAACACATATGCGTCACAAAAA; *actc1a* F: CCAGCACAATGAAGATCAAG and R: CCAGCACAATGAAGATCAAG; *actc1b* F: TGACCGTATGCAGAAGGAGAT and R: TCTTATCACTTATCTGTTT). Imaging was performed with an Olympus SZX16 stereomicroscope.

### cDNA synthesis and quantitative RT-PCR

Total RNA was extracted using TRIzol reagent (Invitrogen Life Technologies). RNA samples were treated with RQ1 RNase-free DNase (Promega). cDNA was synthesized from 1μg of each RNA sample in a 20ml reaction using Protoscript first strand cDNA synthesis kit (New England Biosciences) and oligo(dT)20 primer following the supplier’s instructions. Quantitative PCR was performed on a Roche Lightcycler instrument and normalized against *β-actin* and *RPS18* [29] as reference genes. Primers for quantitative PCR are listed in S3 Table.

### Phylogenetic and synteny analyses of actin isoforms

The human actin protein sequences were used as a query against representative databases from mouse, chicken and zebrafish genomes using a BLASTp search. Corresponding orthologues to all six actin isoforms were identified and aligned using ClustalX [30]. After using the MEGA 5.05 [31] program to determine the best–fit model for the analysis, a neighbor-joining tree (JTT, bootstrapping = 1000) was compiled using MEGA 5.05 [31]. A yeast ACT1 (Genbank ID: 850504) protein sequence was used as an outgroup. To analyze *ACTA1* and *ACTC1* synteny, orthologous genes were identified in Ensembl (http://asia.ensembl.org/index.html) and the flanking genomic regions were annotated.

### Locomotion analyses

Locomotion assays were performed on 6 dpf zebrafish as per [32]. An inactivity threshold of 6 mm/s, detection threshold of 25 mm/s and maximum burst threshold of 30 mm/s were used. The total distance swum above the inactivity threshold and below maximum burst threshold in a 10-min period were extracted using the ZebraLab software (ViewPoint Life Sciences). Blinding of treatments groups was used in combination with randomization of experimental replicates to remove any bias. Once the testing and genotyping was completed the treatments groups were uncovered.

### Western blot and immunofluorescence

Immunofluorescence was performed on 2 dpf zebrafish as per [14] using an anti-Actinin2 antibody (Sigma clone A7811, 1:200), AlexaFluorTM-labelled-596 secondary antibody (Molecular Probes, 1:200) and rhodamine-tagged phalloidin (Molecular Probes, 1:200). For phenotypic experiments, samples were blinded during analyses and genotypes were revealed one all samples were scored. For western blot assays, the head and tails were separated from 2 dpf zebrafish from each condition for three independent biological replicates. The heads were used for genotyping and 20 tails per condition were used for protein lysates as per [33] and quantified using the Qubit fluorometric quantification (Thermo Fisher Scientific). 10–20μg of each sample, along with reducing agent (Life Technologies) and protein loading dye (Life Technologies), was heated at 70°C for 10 min and separated by SDS-PAGE on NuPAGE 4–12% Bis-Tris gels. The protein was transferred onto PVDF membrane (Millipore), following which, the membrane was blocked with 5% skimmed milk in PBST and subsequently probed with anti-Actin (Sigma, A2066, 1/1000), washed and incubated with HRP-conjugated secondary antibody (Southern Biotech, 1:10 000). Immunoblots were developed using ECL prime (GE healthcare) and imaged using a chemiluminescence detector (Vilber Lourmat). The membrane was subsequently stripped, reprobed with anti-β-tubulin antibody (Abcam, ab6046, 1/5000), incubated in HRP-conjugated secondary antibody and developed as above. The membrane was subsequently stripped and stained with Direct Blue 71 (Sigma) to identify total protein. Fiji was used to quantify protein intensity.

### Statistics

For swimming analyses, all values were normalized to the average *actc1b*^*+/+*^ siblings injected with a control MO, or *actc1b*^*+/+*^ siblings. Normality of data was determined using a D’Agostino and Pearson test for normality. Normal data (Fig 2B) was analysed by one-way ANOVA using Dunnett’s correction for multiple comparisons. For data failing the normality test (Fig 2C and Fig 3C), the test was repeated after the outliers were removed by the ROUT method (Q = 1%) or the data was logtransformed. In neither case did this result in a normal distribution of data. Therefore, in these cases the data from the three replicates was pooled and a Kruskal-Wallis test was performed and correction for multiple comparisons conducted using Dunn’s test. For phenotypic analyses (Fig 3B and Fig 5), the results of the three replicates were used to determine the mean percentage of each phenotype and to plot the graphs. The proportion of the phenotypes was determined by pooling the data from all three replicates and conducting a Chi-square test for each treatment against its respective control. For qRT-PCR data (Fig 4C and 4D) a one-way ANOVA was conducted for each gene comparing *actc1b*^*+/-*^ and *actc1b*^*-/-*^ to *actc1b*^*+/+*^ or UTR MO and ex2 MO to Standard Control MO using Dunnett’s test for multiple comparisons. All statistical analyses were conducted using GraphPad Prism 7.

## Supporting information

S1 Fig *In situ* hybridization of *acta1* and *actc1* genes during zebrafish embryogenesis. *acta1a* is expressed in the trunk skeletal muscle (arrows) at 24 hpf (A, A’), 48 hpf (B, B’) and 72 hpf (C, C’) and in the ocular muscle (o), developing pectoral fins (p), fin folds (f), hypaxial muscle (hy) and in the head musculature at 72 hpf (C, C’). *acta1b* is expressed in the heart (arrowheads) and trunk skeletal muscle (arrows) at 24 hpf (D, D’), 48 hpf (E, E’) and 72 hpf (F, F’). *actc1a* is expressed in the heart (arrowheads) and skeletal muscle (arrows) at 24 hpf (G, G’) and 48 hpf (H, H’), with expression in the skeletal muscle localized to the outer edges of the trunk muscle at 72 hpf (I, I’). *actc1a* is expressed in the ocular muscles (o), developing pectoral fins (p), fin folds (f), hypaxial muscle (h) and in the head musculature at 72 hpf (I, I’). *actc1b* is expressed in the heart (arrowheads) and trunk skeletal muscle (arrows) at 24 hpf (J, J’), 48 hpf (K, K’) and in the trunk skeletal muscle at 72 hpf (L, L’). *actc1b* is also expressed in the ocular muscles (o), developing pectoral fins (p), hypaxial muscle (h) and in the head musculature (h) at 72 hpf (L, L’).
(TIF)

S2 Fig Microsynteny analysis of ACTA1 and ACTC1. Comparison of the surrounding genes regions of *Homo sapiens* (human) A) *ACTA1* and B) *ACTC1* and *Danio rerio* (zebrafish) *acta1a*, *acta1b*, *actc1a*, and *actc1b* genes. Orthologous genes are shown in the same colors and connected by dashed lines.
(TIF)

S3 Fig Phylogenetic analysis of ACTA1 and ACTC1. Neighbor joining phylogenetic analysis of ACTA1 and ACTC1 protein sequences from human, mouse, chicken, zebrafish and *Xenopus* (frog) genomes. The tree represents 1000 bootstrapping replicates. The yeast ACT1 protein sequence was used as an outgroup. The duplication giving rise to the ACTA1 and ACTC1 clades is marked by an arrow.
(TIF)

S4 Fig Characterization of the *actc1b^sa12367^* mutant strain. **A)** RT-PCR analysis of *actc1b* mRNA in *actc1b*^*-/-*^ (-/-) mutants compared to their wildtype siblings (*actc1b*^*+/+*^ (+/+) and *actc1b*^*+/-*^ (+/-)). B) Sequencing trace file illustrating the G to T transition (arrow) in *actc1b*^*+/+*^ *actc1b*^*-/-*^ respectively which produces a stop codon (*) as illustrated by the amino acids below the chromatograms. C) Alignment of 5’ region of *actc1b* from *actc1b*^*+/+*^ and *actc1b*^*-/-*^ spanning the 5’UTR and part of intron 2 illustrating that the UTR and ex2 splice MO binding sites are intact in *actc1b*^*-/-*^ mutants.
(TIF)

S5 Fig Western blot expression analyses of α-actin following MO injection. A) Western blot analyses for α-actin protein expression in *actc1b*^*-/-*^ and their wildtype siblings (*actc1b*^*+/-*^ and *actc1b*^*+/+*^) at 2 dpf injected with either an Actc1b UTR, Actc1b ex2 or Standard Control MO for three independent replicate experiments, comprising 20 tails. β-tubulin was used as a loading control. B) Quantification of western blot analysis from three independent replicate experiments from A) whereby α-actin protein levels were normalized against the β-tubulin loading control.
(TIF)

S6 Fig Analysis of Actc1a MO phenotype. A) RT-PCR analysis for *actc1a* following Actc1a MO knockdown. The lower band (arrow) is the expected RT-PCR product of 214bp appearing in both Actc1a MO injected and uninjected embryos. The upper band (arrowhead) appears in the Actc1a MO injected embryos, becoming more apparent as the MO concentration increases, and represents the inclusion of intron 2 resulting from mis-splicing at the exon1/intron2 boundary. B) Western blot analysis and C) quantification of α-actin protein expression in wildtype zebrafish at 2 dpf resulting from increasing doses of Actc1a MO or Standard Control MO, comprising 25 whole embryos. α-actin protein levels were normalized against the β-tubulin loading control. D) Brightfield and cascade blue images of 2 dpf zebrafish embryos injected with increasing doses of Actc1a MO showing the appearance of a dilated heart (arrowheads) in 1.0ng and 2.0ng morphants compared to uninjected controls. Cascade blue was used to identify MO-injected embryos. E) Actinin2 staining of the trunk muscle at 2 dpf reveals no abnormalities in Actc1a MO injected embryos compared to uninjected controls.
(TIF)

S1 Table Delta CT values for qRT-PCR analyses on head samples (see Fig 1). (DOCX)

S2 Table Delta CT values for qRT-PCR analyses on tail samples (see Fig 1). (DOCX)

S3 Table Primers sequences used for qRT-PCR analyses. (DOCX)
