# Supplementary figures and images for: Genetic compensation triggered by actin mutation prevents the muscle damage caused by loss of actin protein
Source: PLoS Genet. 2018 Feb 8;14(2):e1007212. doi: 10.1371/journal.pgen.1007212 (PMC5821405; doi:10.1371/journal.pgen.1007212)

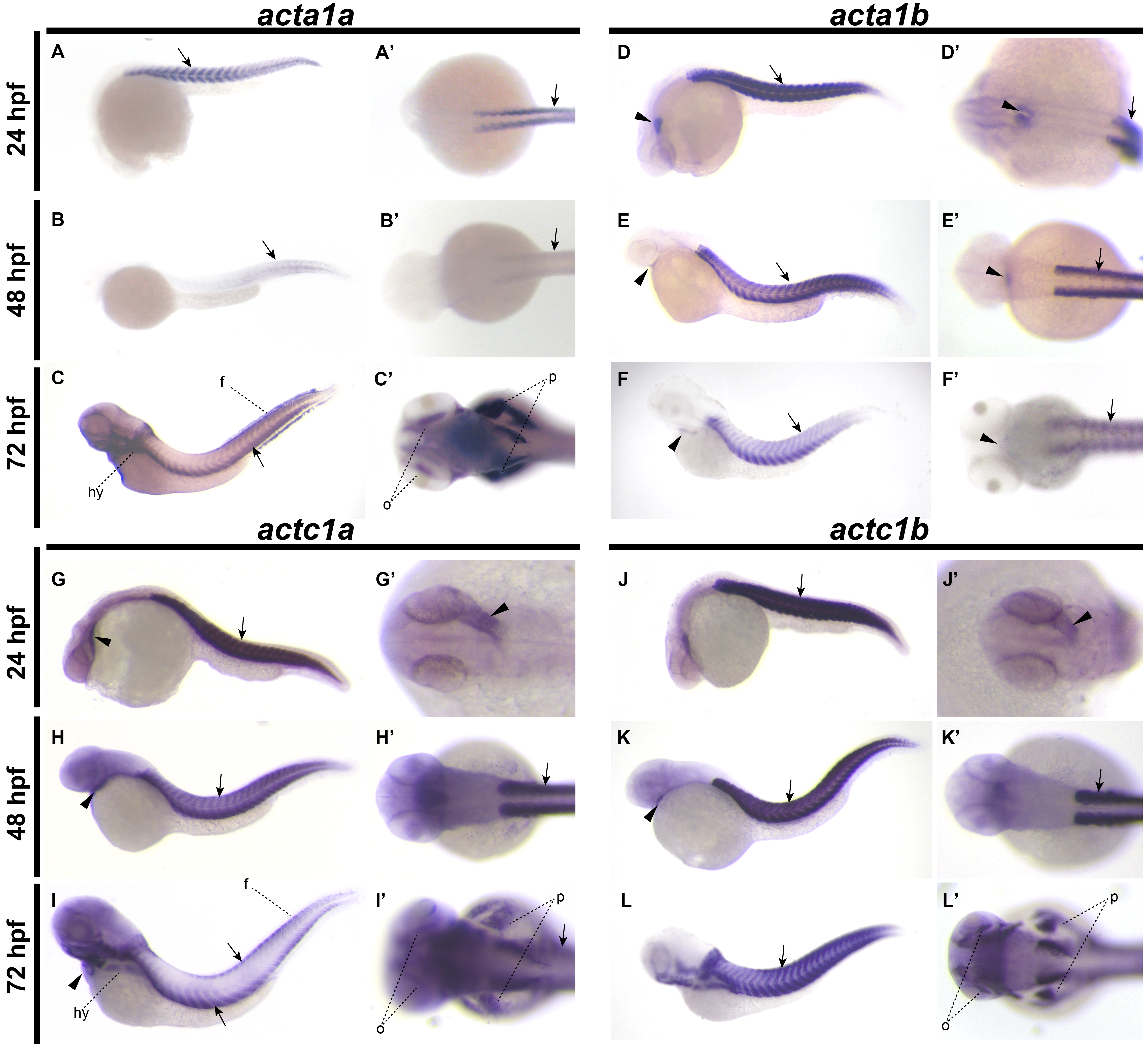

Supplement: S1 Fig — acta1a is expressed in the trunk skeletal muscle (arrows) at 24 hpf (A, A’), 48 hpf (B, B’) and 72 hpf (C, C’) and in the ocular muscle (o), developing pectoral fins (p), fin folds (f), hypaxial muscle (hy) and in the head musculature at 72 hpf (C, C’). acta1b is expressed in the heart (arrowheads) and trunk skeletal muscle (arrows) at 24 hpf (D, D’), 48 hpf (E, E’) and 72 hpf (F, F’). actc1a is expressed in the heart (arrowheads) and skeletal muscle (arrows) at 24 hpf (G, G’) and 48 hpf (H, H’), with expression in the skeletal muscle localized to the outer edges of the trunk muscle at 72 hpf (I, I’). actc1a is expressed in the ocular muscles (o), developing pectoral fins (p), fin folds (f), hypaxial muscle (h) and in the head musculature at 72 hpf (I, I’). actc1b is expressed in the heart (arrowheads) and trunk skeletal muscle (arrows) at 24 hpf (J, J’), 48 hpf (K, K’) and in the trunk skeletal muscle at 72 hpf (L, L’). actc1b is also expressed in the ocular muscles (o), developing pectoral fins (p), hypaxial muscle (h) and in the head musculature (h) at 72 hpf (L, L’). (TIF) [file pgen.1007212.s001.tif]

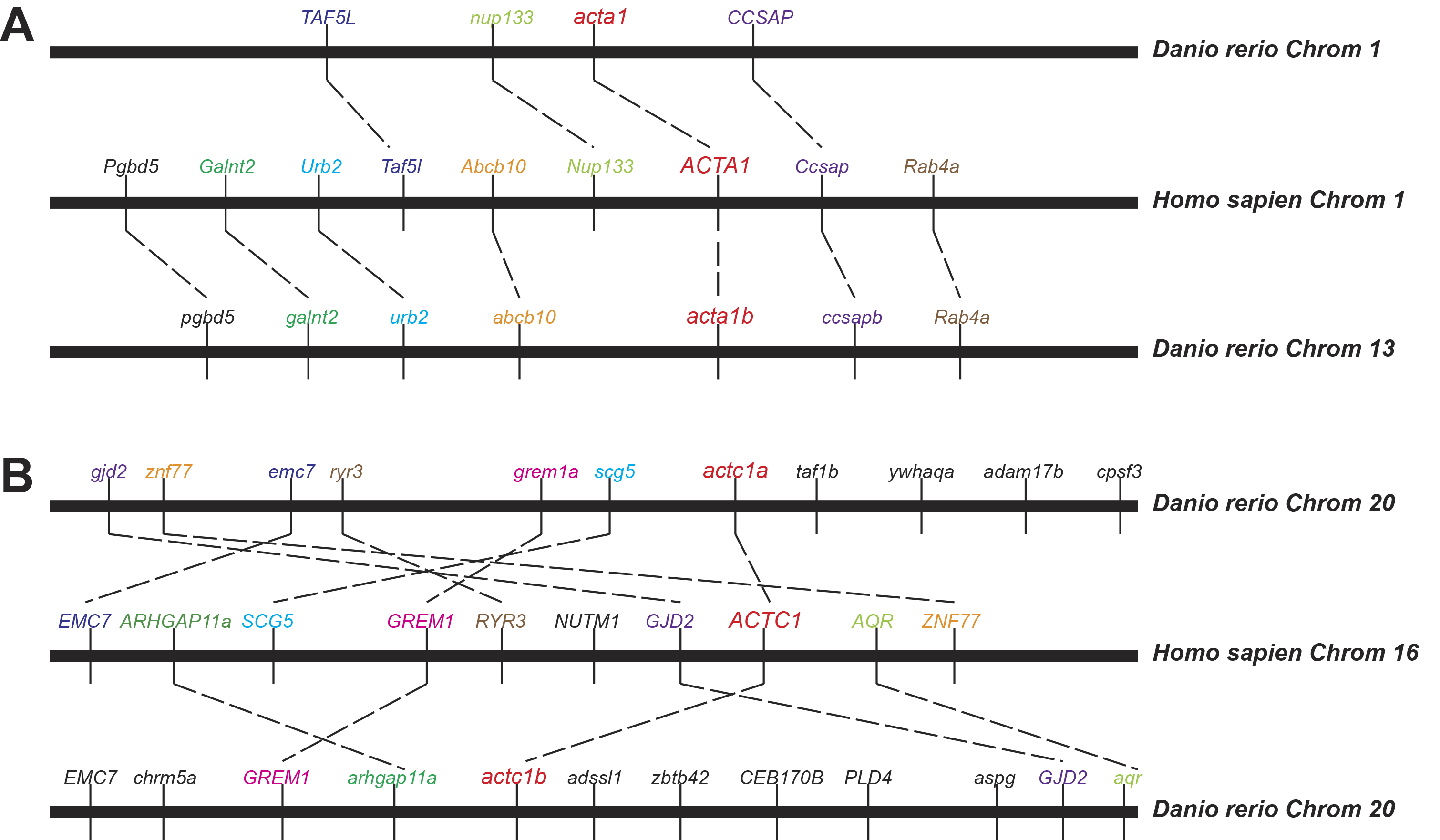

Supplement: S2 Fig — Comparison of the surrounding genes regions of Homo sapiens (human) A) ACTA1 and B) ACTC1 and Danio rerio (zebrafish) acta1a, acta1b, actc1a, and actc1b genes. Orthologous genes are shown in the same colors and connected by dashed lines. (TIF) [file pgen.1007212.s002.tif]

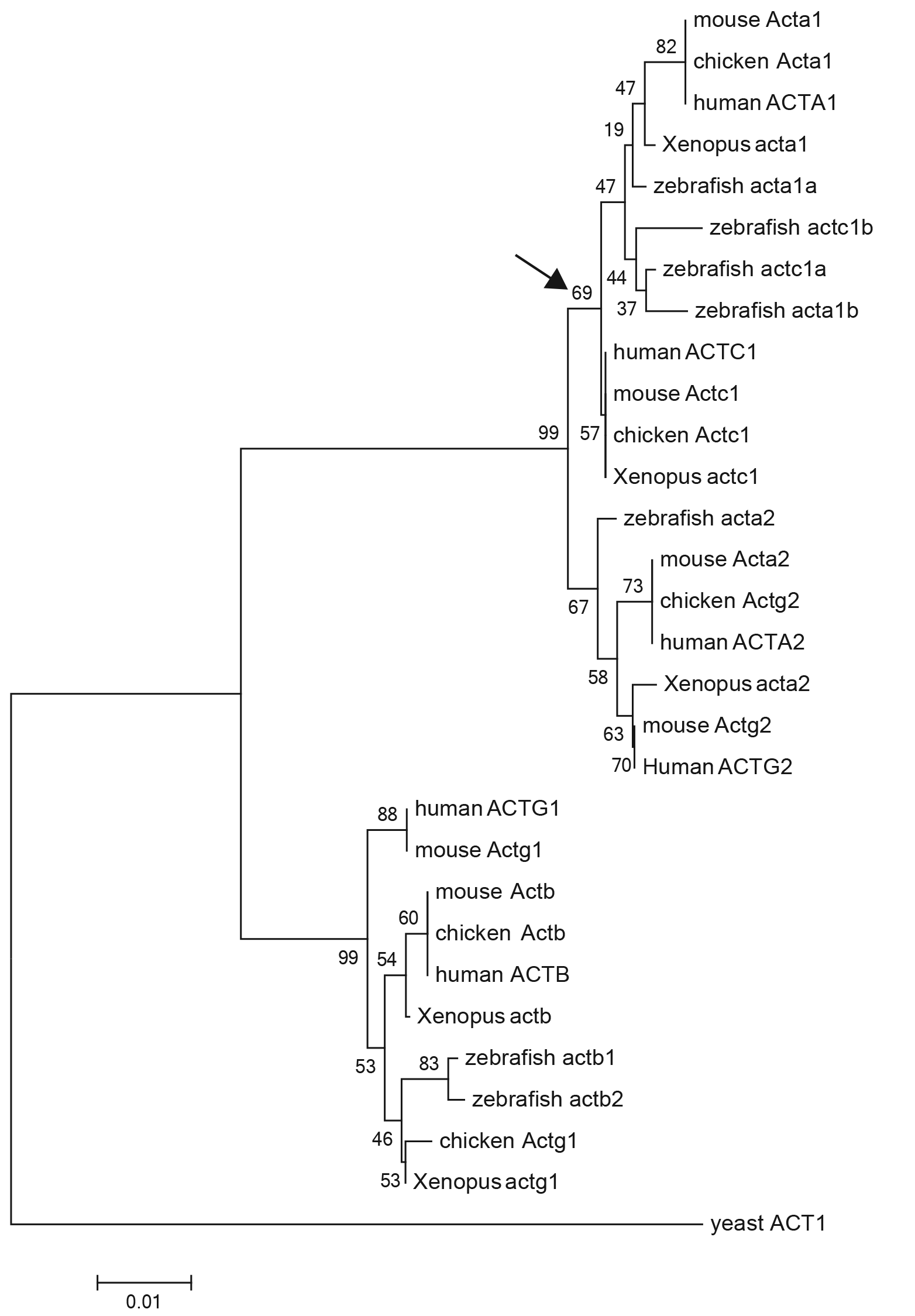

Supplement: S3 Fig — Neighbor joining phylogenetic analysis of ACTA1 and ACTC1 protein sequences from human, mouse, chicken, zebrafish and Xenopus (frog) genomes. The tree represents 1000 bootstrapping replicates. The yeast ACT1 protein sequence was used as an outgroup. The duplication giving rise to the ACTA1 and ACTC1 clades is marked by an arrow. (TIF) [file pgen.1007212.s003.tif]

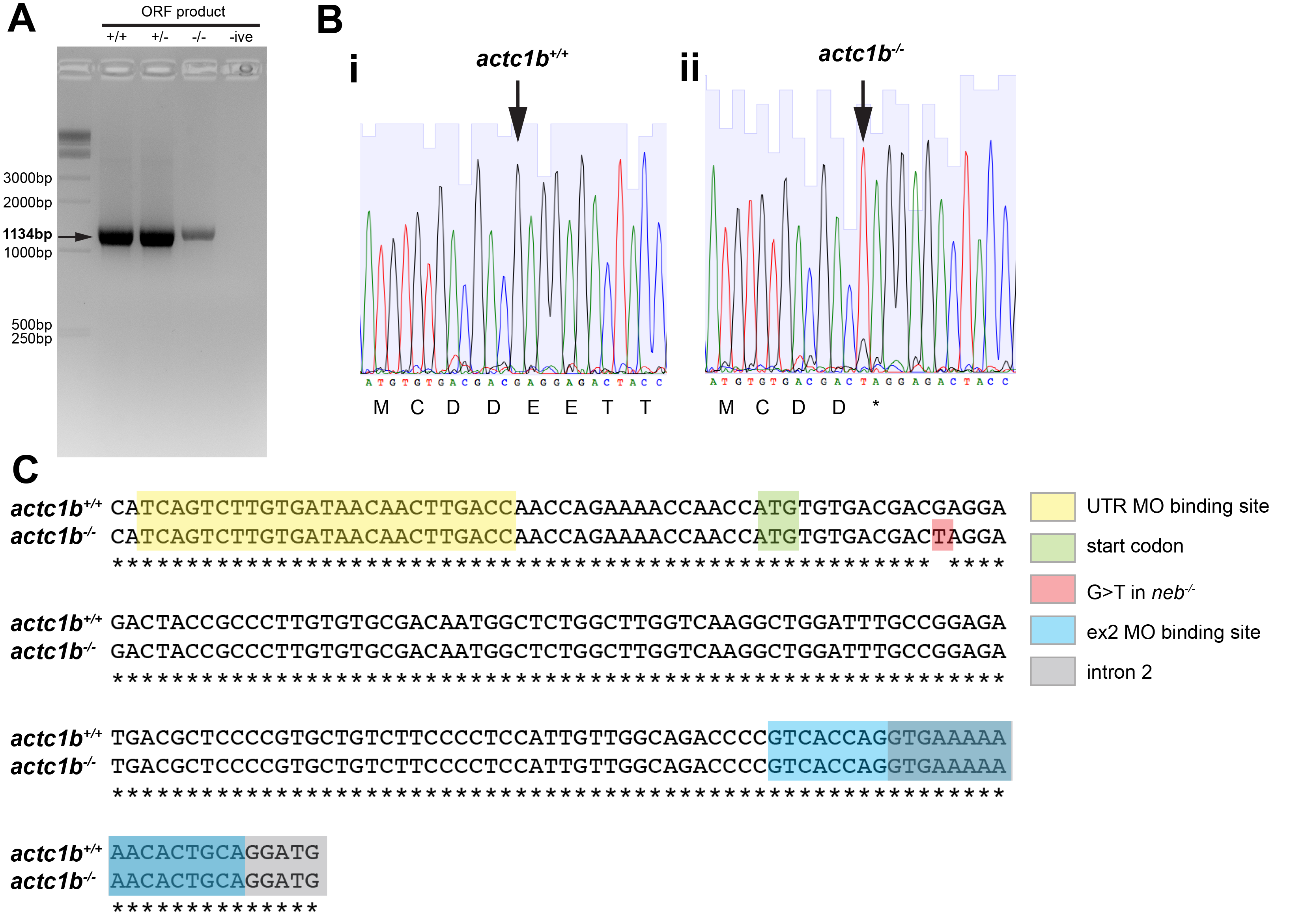

Supplement: S4 Fig — A) RT-PCR analysis of actc1b mRNA in actc1b-/- (-/-) mutants compared to their wildtype siblings (actc1b+/+ (+/+) and actc1b+/- (+/-)). B) Sequencing trace file illustrating the G to T transition (arrow) in actc1b+/+ actc1b-/- respectively which produces a stop codon (*) as illustrated by the amino acids below the chromatograms. C) Alignment of 5’ region of actc1b from actc1b+/+ and actc1b-/- spanning the 5’UTR and part of intron 2 illustrating that the UTR and ex2 splice MO binding sites are intact in actc1b-/- mutants. (TIF) [file pgen.1007212.s004.tif]

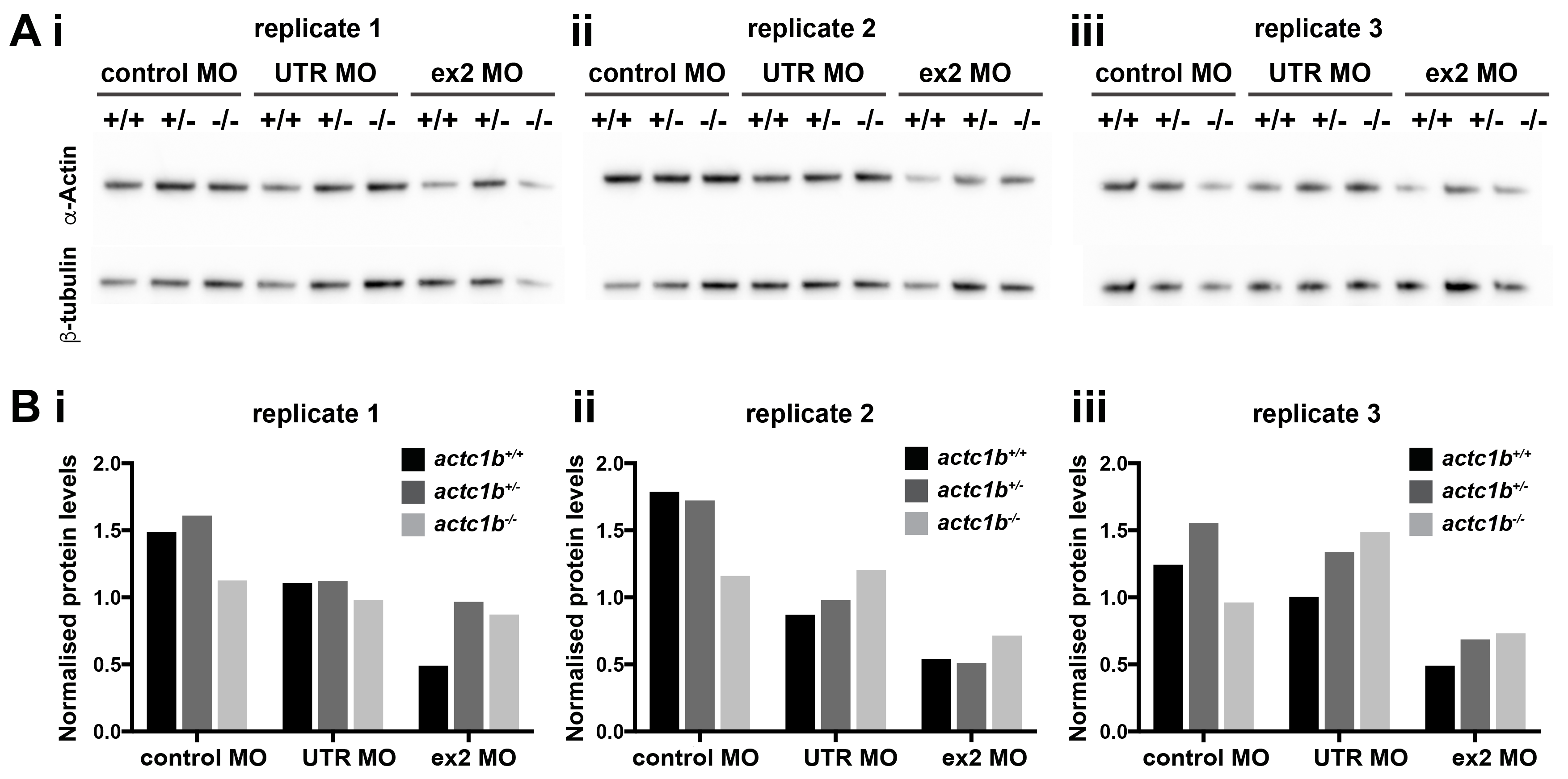

Supplement: S5 Fig — A) Western blot analyses for α-actin protein expression in actc1b-/- and their wildtype siblings (actc1b+/- and actc1b+/+) at 2 dpf injected with either an Actc1b UTR, Actc1b ex2 or Standard Control MO for three independent replicate experiments, comprising 20 tails. β-tubulin was used as a loading control. B) Quantification of western blot analysis from three independent replicate experiments from A) whereby α-actin protein levels were normalized against the β-tubulin loading control. (TIF) [file pgen.1007212.s005.tif]

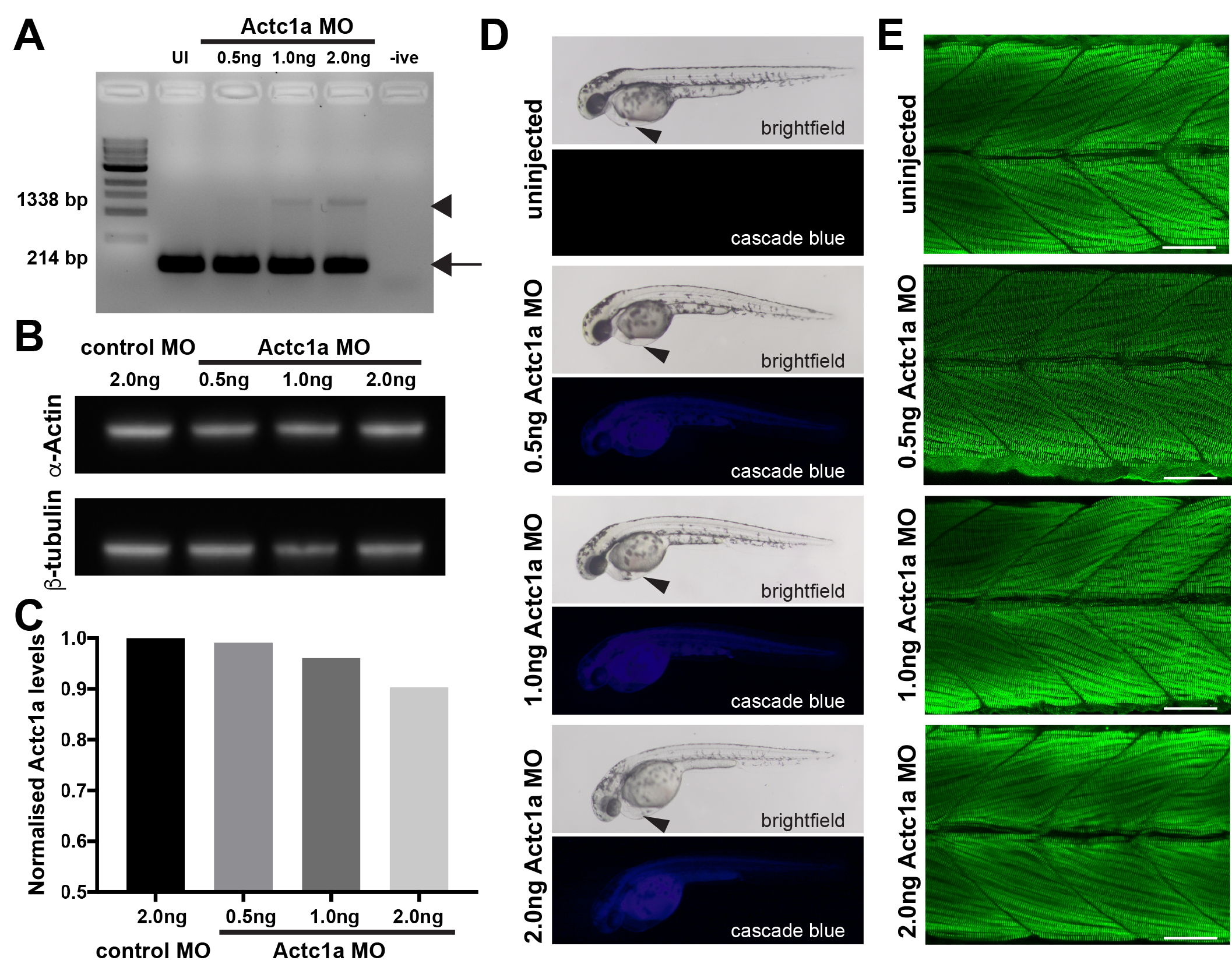

Supplement: S6 Fig — A) RT-PCR analysis for actc1a following Actc1a MO knockdown. The lower band (arrow) is the expected RT-PCR product of 214bp appearing in both Actc1a MO injected and uninjected embryos. The upper band (arrowhead) appears in the Actc1a MO injected embryos, becoming more apparent as the MO concentration increases, and represents the inclusion of intron 2 resulting from mis-splicing at the exon1/intron2 boundary. B) Western blot analysis and C) quantification of α-actin protein expression in wildtype zebrafish at 2 dpf resulting from increasing doses of Actc1a MO or Standard Control MO, comprising 25 whole embryos. α-actin protein levels were normalized against the β-tubulin loading control. D) Brightfield and cascade blue images of 2 dpf zebrafish embryos injected with increasing doses of Actc1a MO showing the appearance of a dilated heart (arrowheads) in 1.0ng and 2.0ng morphants compared to uninjected controls. Cascade blue was used to identify MO-injected embryos. E) Actinin2 staining of the trunk muscle at 2 dpf reveals no abnormalities in Actc1a MO injected embryos compared to uninjected controls. (TIF) [file pgen.1007212.s006.tif]

**Supplementary Table 1**: Delta CT values for qRT-PCR analyses on head samples (see Figure 1).


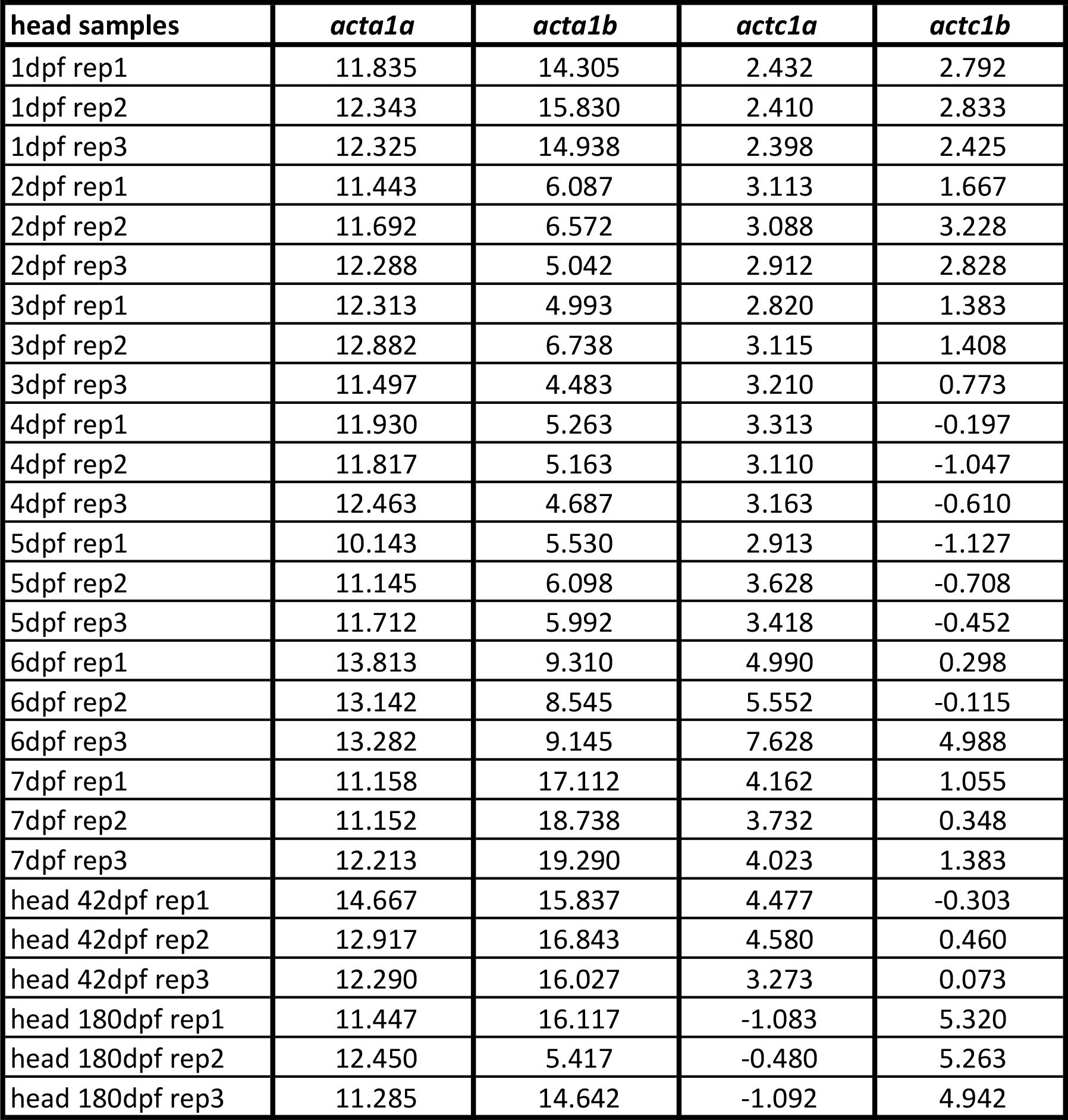

Supplement: S1 Table — (DOCX) [file pgen.1007212.s007.docx]

**Supplementary Table 2**: Delta CT values for qRT-PCR analyses on tail samples (see Figure 1).


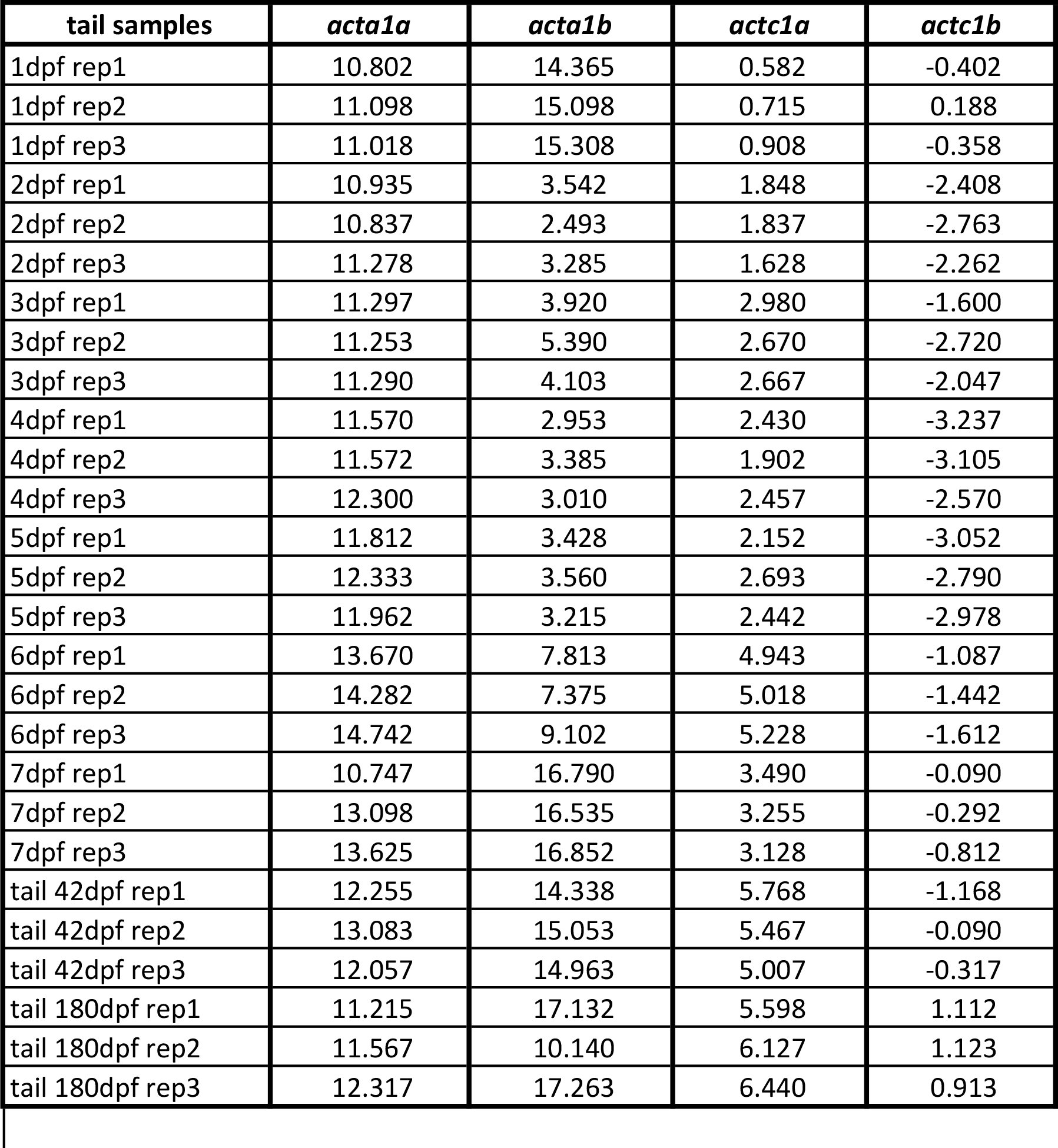

Supplement: S2 Table — (DOCX) [file pgen.1007212.s008.docx]
