# Supplementary material for: Genetic compensation triggered by actin mutation prevents the muscle damage caused by loss of actin protein
Source: PLoS Genet. 2018 Feb 8;14(2):e1007212. doi: 10.1371/journal.pgen.1007212 (PMC5821405; doi:10.1371/journal.pgen.1007212)
Supplement: S3 Table — (DOCX) [file pgen.1007212.s009.docx]

**Supplementary Table 3**: Primers sequences used for qRT-PCR analyses.

| **Gene** | **Primer Sequence** | **Orientation** |
| --- | --- | --- |
| *β-actin* | GCATTGCTGACCGTATGCAG | forward |
| *β-actin* | GATCCACATCTGCTGGAAGGTGG | reverse |
| *RPS18* | TCGCTAGTTGGCATCGTTTATG | forward |
| *RPS18* | CGGAGGTTCGAAGACGATCA | reverse |
| *acta1a* | AAAGCAGAGGACGGGTTTGT | forward |
| *acta1a* | GTTCTGCCTCTGCTCGTTCT | reverse |
| *acta1b* | ATTCATCGGCTGCATCTGTC | forward |
| *acta1b* | CCCAAAGCTGTCCCATAATTT | reverse |
| *actc1a* | CAGCGGAAACTCCAGTCTTG | forward |
| *actc1a* | CTGACCCATACCAACCATCA | reverse |
| *actc1b* | TCCCAGTGGAAGCATCAGTC | forward |
| *actc1b* | TGGGGTACTTCAGGGTCAAG | reverse |
